# Association Between Kidney Stone and Dental Calculus in a Saudi Population: A Cross-Sectional Study

**DOI:** 10.7759/cureus.37279

**Published:** 2023-04-08

**Authors:** Hassan H Kaabi, Rita M Khounganian, Mohammad A Alomar, Rayan A Ali Al-qarni, Saad G Alshaiban, Sultan M Aljumah, Moath A Alzahrani, Abdulmosen A Alanazi, Ahmed Almslam, Atif A Alghamdi

**Affiliations:** 1 Department of Oral Medicine and Diagnostic Sciences, College of Dentistry, King Saud University, Riyadh, SAU; 2 Department of Urology, King Faisal Specialist Hospital and Research Centre, Riyadh, SAU; 3 College of Dentistry, King Saud University, Riyadh, SAU

**Keywords:** plaque index, calculus index, dental plaque, dental calculus, kidney stone

## Abstract

Objective: To investigate the correlation between dental calculus and kidney stones, and to identify the risk factors associated with the presence of these conditions.

Methods: This study was carried out at the medical city, King Saud University, Riyadh, Saudi Arabia between 2020 and 2021. The study included 141 participants (70 with kidney stones and 71 with controls). The dental plaque and calculus indices were used to record plaque and calculus scores, respectively. All information was statistically investigated and the level of significance was set at *p*<0.05.

Results: The plaque and calculus indices were significantly higher in the control group when compared to the kidney stone group (*p*<0.05). A weak positive correlation between age and the calculus index in the kidney stone group was revealed (*r*=0.31, *p*=0.01). However, only within the age group 36-55, the results showed that the control group had a significantly higher calculus index than that of the kidney stone group (*p*=0.02). The married patients with kidney stones scored a significantly higher plaque index than the unmarried patients (*p*=0.03).

Conclusion: The dental plaque and calculus indices were lower in the kidney stone group than those of the non-kidney stone group. Therefore, the clinical observation of dental plaque and calculus may not be indicators of kidney stones. However, within the kidney stone group, elderly and married patients could be at a higher risk for developing dental calculus and plaque, respectively.

## Introduction

Dental calculus or tartar is a form of calcified dental plaque, which is caused by the mineralization of plaque on tooth surfaces. The minerals forming dental calculus are mainly saliva and gingival crevicular fluid. Bacterial cells within the dental plaque are destroyed by such mineralization, however, the roughness of the mineralized plaque is an ideal nest for further bacterial growth leading to calculus build-up [1]. Calculus accommodating an unmineralized layer of viable bacterial plaque is a predisposing factor for compromising the health of periodontal tissues [2].

Dental calculus is classified into supragingival (along the gum line) and subgingival (between the tooth and gum) [3]. Dental calculus consists of both minerals (inorganic phase 40-60%) and cellular and extracellular matrix (organic phase) [1]. Mineralized biofilms consist of brushite, whitlockite, octacalcium phosphate, and hydroxyapatite. The composition and amount of calculus depend on the composition of the saliva and the location of the calculus formed (supragingival vs subgingival) [4,5]. Interestingly, dental calculus is similar to all biological systems, including kidney stones, in terms of structural composition and mineralization process [6].

Kidney stones, also known as renal stones or nephrolithiasis, are hard deposits made of minerals and salts that form inside kidneys and are the most common urinary tract disease. Its prevalence and recurrence rates are increasing in developing and developed nations, and it may affect approximately 12% of the world population [7]. The four major types of renal calculi include calcium, uric acid, struvite, and cystine stones [8].

Body stones may be a part of a systemic biomineralization process [9], and a significant amount of research has been carried out to understand such stones. Nevertheless, only limited reports shed light on the association between calcifications that occur in different organs of the same individual. The aim of our baseline study was to investigate the correlation between dental calculus and kidney stones and to identify the associated risk factors.

## Materials and methods

This study was carried out at two different centers (King Khalid Hospital and Dental University Hospital), King Saud University, Riyadh, Saudi Arabia, following the approval of the Institutional Review Board of King Saud University Medical City (IRB No. #E-20-5199) and the College of Dentistry Research Centre (CDRC No. #IR0366). The sample size needed for the conduction of this study was calculated using G*Power software (Franz Faul, Universitat Kiel, Germany). Using an effect size of 0.25 and power of 0.9, a type I error (α) set at 0.05, the total sample size should be a minimum of 65 per group. This number was exceeded as the study was carried out with 141 participants for both groups.

To determine the correlation between dental calculus and kidney stones, the participants were divided into two groups. The first group (study group) included a random sample of 70 patients who were diagnosed with kidney stones from the Endo-urology clinic of Dr. Alomar at King Khalid Hospital. The second group (controls) consisted of 71 individuals randomly selected from the patients visiting the clinics of the Dental University Hospital, who had no history of kidney stones. Informed consent was obtained from all participants during the study.

We collected the medical and dental histories of all the participants. The study included all participants who practiced regular tooth brushing and had no history of any systemic diseases. Those who have undergone calculus removal within the last three months or who had any prosthetic or orthodontic appliances were excluded. Patients without a history of kidney stones were included in the control group, while those with kidney stones were included in the study group.

The plaque index and calculus index were used to assess the amount of dental plaque and calculus, respectively. The amount of plaque or calculus was measured from three upper and lower teeth according to a previously described protocol [10].

To reduce the possibility of bias during the data collection, the inter- and intra-examiner reliability was tested using the Kappa and Pearson correlation coefficient (r). The agreement between the examiners on average was Kappa>0.7 and r=0.81. Moreover, the agreement between the first examination and re-examination performed one hour later by the same investigator was on average Kappa>0.85 and r=0.9.

Descriptive statistics, frequency, percentages, mean, and standard deviation are presented. A normal distribution was assumed since the sample size in each group was >30 using the central limit theorem. For statistical inferences, an independent t-test was used for measuring the average difference between the two groups. In addition, Pearson correlation coefficients were employed for measuring the correlation between the numerical variables (index and age). The assumption of regression analysis was not met, so other statistical techniques for the risk factors were used for accuracy. The level of significance was set at α=0.05; thus, a p-value<0.05 was considered statistically significant. Collected data were analyzed using the Statistical Package for the Social Sciences (SPSS) software version 26.0 (IBM Inc., Armonk, NY, USA).

## Results

The present study included 141 patients; 71 (50.4%) were in the control group and 70 (49.6%) had kidney stones. The age of the patients ranged from 18 to 74 with a mean age of 38.4±13.9 years. In addition, 92 (65.2%) patients were male, and 49 (34.8%) were female. Kidney stones were more prevalent among males (73%) than females (27%), and widespread within the group aged 36-55 years. The demographic data are presented in Table 1.

**Table 1:** Demographic data Values are presented as the total number and (%).

Characteristics		Control	Kidney stone
		71 (50.4)	70 (49.6)
Gender	Male	41 (58)	51 (73)
	Female	30 (42)	19 (27)
Age group	18-35	41 (57.8)	25 (35.7)
	36-55	26 (36.6)	30 (42.9)
	≥56	4 (5.6)	15 (21.4)
Marital status	Unmarried	35 (49.3)	11 (15.7)
	Married	36 (50.7)	59 (84.3)
Smoking	Yes	9 (12.7)	14 (20)
	No	62 (87.3)	56 (80)

The calculus and plaque indices were significantly higher in the control group when compared to the kidney stone group (*p*<0.05) (Table 2).

**Table 2:** Difference between control and kidney stone groups according to the plaque and calculus indices. *Statistically significant difference (*p*<0.05). SD: standard deviation.

Index	Mean ± SD		*P*-value
	Control	Kidney stone	
Calculus index	1.15 ± 0.55	0.92 ± 0.54	0.02^*^
Plaque index	1.40 ± 0.49	1.21 ± 0.54	0.03^*^

The results revealed a weak positive correlation between age and the calculus index (*r*= 0.31, *p*=0.01) in the kidney stone group, whereas the plaque index showed a weak positive correlation (*r*=0.32, *p*=0.05). Moreover, there was no significant correlation between age and the indices for the control group (Table 3).

**Table 3:** Correlation between age and indices. *r*: Pearson correlation coefficient test, *Statistically significant difference (*p*<0.05).

Index	Control (71)		Kidney stone (70)	
	r	P-value	r	*P*-value
Calculus index	0.04	0.74	0.31	0.01*
Plaque index	0.16	0.19	0.23	0.05

The effects of different age groups on the amount of calculus and plaque were investigated (Table 4). Overall, all age groups had higher calculus and plaque indices in the control group when compared to the kidney stone group but the difference was not statistically significant. However, only within the age group 36-55, the independent t-test showed that the control group had a significantly higher calculus index than the kidney stone group (*p*=0.02).

**Table 4:** Difference between the control and kidney stone patients within each age group for calculus and plaque indices. *Statistically significant difference (*p*<0.05). SD: standard deviation.

Age group	Index	Mean ± SD		*P*-value
		Control	Kidney stone	
18-35 years old (n=66)	Calculus index	1.05 ± 0.52	0.80 ± 0.44	0.05
	Plaque index	1.31 ± 0.42	1.11 ± 0.50	0.09
36-55 years old (n=66)	Calculus index	1.29 ± 0.53	0.94 ± 0.55	0.02*
	Plaque index	1.50 ± 0.56	1.25 ± 0.49	0.08
≥56 years old (n=19)	Calculus index	1.29 ± 0.88	1.10 ± 0.66	0.65
	Plaque index	1.71 ± 0.60	1.31 ± 0.69	0.31

The calculus and plaque indices were higher in males compared to females in both the control and kidney stone patients. However, the differences were not statistically significant, as shown in Table 5. The calculus and plaque indices were further examined separately in the married and unmarried patients (Table 5). The indices were higher in the married patients in both the control and kidney stone groups. However, a statistically significant difference was only observed for the plaque index among married participants in the kidney stone group (*p*=0.03).

**Table 5:** Difference between indices for gender and marital status within control and kidney stone groups. *Statistically significant difference (*p*<0.05). SD: standard deviation.

Group	Index	Mean ± SD		*P*-value
		Male	Female	
Control	Calculus index	1.21 ± 0.59	1.06 ± 0.50	0.27
	Plaque index	1.46 ± 0.50	1.32 ± 0.48	0.26
Kidney stone	Calculus index	0.98 ± 0.58	0.78 ± 0.43	0.17
	Plaque index	1.22 ± 0.52	1.21 ± 0.61	0.99
Group	Index	Unmarried	Married	*P*-value
Control	Calculus index	1.83 ± 0.56	1.86 ± 0.49	0.85
	Plaque index	1.97 ± 0.45	2.17 ± 0.45	0.07
Kidney stone	Calculus index	1.36 ± 0.50	1.73 ± 0.58	0.05
	Plaque index	1.64 ± 0.50	2 ± 0.49	0.03*

## Discussion

The present preliminary baseline research was undertaken to investigate the correlation between dental calculus and kidney stones. Body stones may be a part of a systemic biomineralization process [9] and can form in different parts of the human body like the kidneys, gall bladder, salivary glands, and within the dental pulp, classified as pathological calcifications [11]. Although numerous studies have investigated stones in the body, few have reported the relationship between them.

The calcification of dental plaque is key for forming dental calculus. The calculus acts as a nest for further accumulation of dental plaque, which again calcifies and forms larger stones [12]. The present study demonstrated a positive correlation between the calculus index and the plaque index for the control and kidney stone groups. This is in agreement with previous studies that found a positive correlation between the value of plaque index and calculus formation [13,14].

Contrary to expectations, our results showed that calculus and plaque indices were lower in patients with kidney stones than those in healthy patients, in particular for the age group 36-55. This condition, which is not related to the frequency of the dental calculi, may be directly related to oral hygiene status. This contradicts other studies, which suggested that the formation of dental calculus may be an indicator of other types of stones, such as kidney stones [11,13]. Although caution must be used in interpreting the results due to the small sample size, these findings suggest that the formation of dental plaque and calculus may not predict the development of kidney stones.

The patients were assessed based on gender, age, marital status, oral hygiene, kidney stone history, dental calculus, and smoking habits. The present study demonstrated gender-based differences in terms of plaque and calculus indices. Although not statistically significant, the data showed that males had poorer plaque and calculus indices than females in both kidney stone and non-kidney stone groups (Table 3). This may demonstrate that females pay more attention to oral health and have better oral hygiene habits than males. Research shows that women are more likely to receive professional dental care, comply with recommended oral health treatment, and have higher oral health literacy, thus presenting with lower levels of plaque and calculus than men [15,16].

Dental plaque deposition and calculus accumulation increase with age [17,18]. Similarly, we found higher plaque and calculus indices in older patients compared to younger patients of the kidney stone group (Tables 3-4). A recent study demonstrated varied compliance levels of kidney stone patients to preventive stone practices in general, however, the difference in compliance between the different age groups was not investigated [19]. Younger patients with kidney stones may be more compliant with oral hygiene instructions than older patients, which may be reflected in the lower amount of dental plaque and calculus.

Our data revealed poorer oral hygiene among married patients, in accordance with a previous study [20], which was mainly represented by a higher plaque index in the kidney stone group. This could be attributed to the fact that marriage life comes with more responsibilities at the expense of oral health. In contrast, a similar study found that married men were more likely to receive dental treatment compared to unmarried men [21].

The finding of the current study has some limitations. First, it is a cross-sectional design, which precludes causal inferences about the association between dental calculus and kidney stones. Moreover, the results in this report were limited to one medical city at King Saud University, Riyadh, Saudi Arabia.

## Conclusions

The following baseline study has shown that dental plaque and calculus indices were lower in patients with kidney stones. Henceforth, the presence of dental plaque and calculus may not be useful indicators of kidney stones. However, within the kidney stone group, elderly and married patients could be at a higher risk for developing dental calculus and plaque, respectively. Considerably more exploratory research work to study the diagnostic value of dental calculus as a predictor for systemic stones and the associated risk factors is highly recommended.
